# Maternal, healthcare, and nutritional factors influencing birth defects in China from 2019 to 2025: a systematic review and meta-analysis

**DOI:** 10.3389/fped.2026.1842814

**Published:** 2026-07-03

**Authors:** Min Yang, Jia Lv, Tianzhi Guo, Min Ren, Lingling Xie, Suhua Tu

**Affiliations:** 1 School of Nursing, Southwest Medical University, Luzhou, Sichuan, China; 2 Department of Obstetrics, Affiliated Hospital of Southwest Medical University, Luzhou, Sichuan, China; 3 Department of Obstetrics and Gynecology, Affiliated Hospital of Southwest Medical University, Luzhou, Sichuan, China; 4 Department of Nursing, Affiliated Hospital of Southwest Medical University, Luzhou, Sichuan, China

**Keywords:** birth defect, China, folic acid, influencing factors, maternal age, meta-analysis, prenatal care, systematic review

## Abstract

**Objective:**

This study aims to systematically evaluate the primary influencing factors of birth defects in China from 2019 to 2025, providing evidence for decision-making and intervention measures in the prevention of birth defects.

**Methods:**

A comprehensive search was conducted in various databases, including CNKI, Wanfang, VIP, China Biomedical Literature Database, PubMed, Web of Science, Embase, Elsevier Science Direct, and CINAHL, covering literature published from January 2019 to December 2025. Two researchers independently performed literature screening, quality assessment, and data extraction. Meta-analysis was conducted using Stata 17.0 software. For factors exhibiting high heterogeneity (*I*^2^ ≥ 50%) and with at least four included studies, subgroup analyses were carried out based on the types of birth defects, study regions, or study quality. This review adhered to the PRISMA 2020 guidelines.

**Results:**

A total of 25 studies were included in this analysis, comprising 26,019 cases of birth defects. Seventeen influencing factors were identified. Maternal physiological factors included maternal age ≥35 years (OR = 1.26, 95% CI: 1.05–1.47), maternal BMI > 30 kg/m^2^ (OR = 1.42, 95% CI: 1.25–1.58), and maternal drug use (OR = 1.06, 95% CI: 0.58–1.54). Healthcare service factors encompassed premarital examinations (OR = 0.36, 95% CI: 0.30–0.43), pregnancy examinations (OR = 0.41, 95% CI: 0.21–0.62), and birth examinations (OR = 0.45, 95% CI: 0.28–0.62). Nutritional and behavioral factors included irregular folic acid supplementation (OR = 1.32, 95% CI: 0.09–2.55; for neural tube defects: OR = 2.34, 95% CI: 2.01–2.72, *I*^2^ = 0%), a history of medication in early pregnancy (OR = 1.17, 95% CI: 0.65–1.69), a history of fever in early pregnancy (OR = 1.19, 95% CI: 0.86–1.52), adverse mood during pregnancy (OR = 1.09, 95% CI: 0.17–2.01), exposure to harmful substances (OR = 1.45, 95% CI: 1.04–1.87), and contraceptive use within 6 months prior to pregnancy (OR = 1.36, 95% CI: 1.29–1.44). Other factors included income <5,000 yuan (OR = 1.41, 95% CI: 0.90–1.93), preterm birth (OR = 1.07, 95% CI: 0.04–2.10; stronger for congenital heart disease: OR = 1.82, 95% CI: 1.64–2.02), being small for gestational age (OR = 1.38, 95% CI: 0.74–2.01), adverse pregnancy history (OR = 1.03, 95% CI: 0.58–1.48), and a family history of genetic disorders (OR = 1.10, 95% CI: 0.81–1.39). Subgroup analyses indicated that after categorizing by types of birth defects, the heterogeneity of factors such as adverse pregnancy history, preterm birth, history of fever in early pregnancy, and irregular folic acid supplementation was significantly reduced, resulting in more stable pooled effect sizes.

**Conclusion:**

Numerous factors influence the occurrence of birth defects. Medical professionals should prioritize addressing irregular folic acid supplementation, low rates of premarital examinations, and regional disparities in prenatal care as key intervention targets in China. It is essential to implement targeted interventions on controllable factors to prevent and reduce the incidence of birth defects.

## 1 Introduction

Birth defects, also known as congenital anomalies, are defined as structural or functional abnormalities present at birth. These include conditions such as congenital heart disease, neural tube defects, cleft lip and palate, and limb abnormalities. According to the World Health Organization, birth defects account for approximately 6% of all infants. However, since statistical data often do not consider terminated pregnancies or stillbirths, the actual incidence of birth defects may be significantly higher. This concern is further highlighted in the China Birth Defect Prevention Report (2012) released by the Chinese Ministry of Health (1), the incidence rate of birth defects in China is approximately 5.6%, with about 900,000 new cases of birth defects each year. Recent studies have confirmed a consistently high burden of birth defects, with the average annual incidence remaining around 5.4%–5.8% in large surveillance cohorts from 2017 to 2021 (2–4). Birth defects have emerged as one of the leading causes of early miscarriage, neonatal and infant mortality, as well as childhood disability.

Despite the significant burden of birth defects, existing evidence regarding their risk factors in China has notable limitations. Firstly, the majority of published studies are either single-center or region-specific, characterized by small sample sizes and inconsistent adjustments for confounding variables. Secondly, previous meta-analyses examining risk factors for birth defects in China were published prior to 2020 and failed to account for the period following major policy changes (5–7). Lastly, few systematic reviews have conducted subgroup analyses based on specific types of birth defects, which is essential given that various defects may have distinct etiologies.

In recent years, the full implementation of the “three-child policy” announced in 2021 has led to an increase in the number of women of childbearing age within the advanced maternal age population. This demographic shift has resulted in a higher proportion and number of high-risk pregnant women, which may exacerbate the risk of birth defects (2). This challenge significantly restricts the overall improvement of population quality and the protection of children's health (3, 4), thereby imposing stricter requirements on the quality and efficiency of maternal and child health services. Therefore, an updated systematic review focusing on the period from 2019 to 2025 is urgently needed to provide contemporary evidence for policymakers and clinicians.

This study aims to conduct a comprehensive meta-analysis of case-control studies published between 2019 and 2025 regarding the factors influencing birth defects in China. Additionally, we will perform subgroup analyses based on defect type, region, and study quality to explore sources of heterogeneity and identify risk factors specific to China.

## 2 Materials and methods

### 2.1 Literature inclusion and exclusion criteria

Inclusion criteria: (1) Case-control studies examining the influencing factors of birth defects in China, published both domestically and internationally from 2019 to 2025; (2) Clearly defined case and control groups; (3) Consistent definitions of exposure to influencing factors; (4) Clearly defined sample sizes; (5) Articles providing odds ratios (OR) and 95% confidence intervals (CIs), or allowing conversion to OR and 95% CIs; (6) Clearly defined study periods.

Exclusion criteria: (1) Studies lacking clearly defined sample sizes; (2) Incorrect data analysis methods; (3) Low-quality literature (Newcastle-Ottawa Scale score < 4); (4) Duplicate or review literature; (5) Studies where the population explicitly excludes common types of birth defects.

Justification for the 2019–2025 time frame: The period from 2019 to 2025 was chosen because it encompasses the full implementation of China's three-child policy (announced in 2021), which has resulted in a significant increase in pregnancies among advanced maternal age and high-risk groups. Additionally, national maternal health policies, such as folic acid supplementation programs and prenatal screening guidelines, were updated during this time. Consequently, a focused synthesis of recent evidence is more pertinent to current clinical practice and policymaking than older data.

### 2.2 Literature search strategy

A combination of subject headings and free-text terms was employed to conduct a comprehensive search across both Chinese and English databases, including CNKI, Wanfang, VIP, the China Biomedical Literature Database, the China Academic Journal Full-text Database, PubMed, Web of Science, Embase, Elsevier Science Direct, and CINAHL, for literature regarding the influencing factors of birth defects in China. Additionally, the snowball method was utilized to trace the references of included studies. The final search was conducted on December 31, 2025. Detailed search strings for all databases are provided in Supplementary File S1. This systematic review adhered to the PRISMA 2020 statement, and a completed PRISMA checklist is included as Supplementary Material.

### 2.3 Literature screening and data extraction

In accordance with the research objectives and the established inclusion and exclusion criteria, two researchers independently conducted a literature screening and data extraction process, followed by a cross-check of their findings. Any disagreements were resolved through discussions involving a third researcher. During the literature screening phase, the titles of articles were initially reviewed to exclude those that were clearly irrelevant. This was followed by a more detailed examination of abstracts and full texts to ascertain eligibility for inclusion. Authors of original studies were contacted via email or telephone to acquire crucial information that was not readily available but essential for the current study. Data extraction was carried out using a pre-designed form that captured key details, including the author, publication year, sample size (for both case and control groups), language of publication, study region, and influencing factors.

### 2.4 Literature quality assessment

The Newcastle-Ottawa Scale (NOS) was employed to evaluate the quality of the studies included in this review. This scale consists of three sections—selection, comparability, and exposure—comprising a total of eight items. Each item is associated with specific scoring criteria, culminating in a maximum score of 9 points; higher scores signify superior study quality. Studies achieving scores between 7 and 9 points were categorized as high quality, those with scores from 4 to 6 points were deemed medium quality, and studies scoring below 4 points were classified as low quality (5). Two researchers independently assessed the quality of the included literature, with any disagreements being resolved by a third researcher. The quality scores (NOS) were utilized to inform subgroup analyses when at least four studies were available for a particular factor (e.g., adverse mood during pregnancy was stratified by NOS scores of 7 vs. 8).

### 2.5 Statistical methods

Statistical analysis was conducted using Stata 17.0. (1) Heterogeneity Test: A fixed-effects model was employed for pooling when *P* > 0.05 and *I*^2^ < 50%. Conversely, when *I*^2^ ≥ 50%, indicating high heterogeneity, a random-effects model was selected. The final pooled results were primarily derived from the random-effects model, with the fixed-effects model results serving as a reference for sensitivity analysis. (2) Subgroup Analyses: For factors exhibiting high heterogeneity (*I*^2^ ≥ 50%) with at least four included studies, further subgroup analyses were performed to explore the sources of heterogeneity. Subgroup classifications included types of birth defects (e.g., congenital heart disease, neural tube defects, cleft lip and palate), study region (southern/northern China), and study quality (NOS score). It is important to note that when the number of studies within a subgroup was limited (<3), the pooled results were considered exploratory and not definitive conclusions. (3) Publication Bias Assessment: For factors with a minimum of ten included studies, publication bias was evaluated using funnel plots in conjunction with Egger's test; a *P*-value >0.05 indicated no significant publication bias. For factors with fewer studies (<10), no formal publication bias test was conducted, and only descriptive interpretations were provided. (4) Sensitivity Analysis: The method of altering the statistical effect model was employed, comparing odds ratios (OR) to assess the stability of results (6). To systematically address heterogeneity across different types of birth defects, we pre-specified subgroup analyses by defect type (congenital heart disease, neural tube defects, cleft lip and palate, and others) for any risk factor that had at least four studies reporting the factor and where the defect type could be clearly identified from the original study. The results of these defect-specific subgroup analyses are presented in Supplementary Table S1 and summarized in the Results section.

## 3 Results

### 3.1 Literature screening and quality assessment

**Figure 1:**
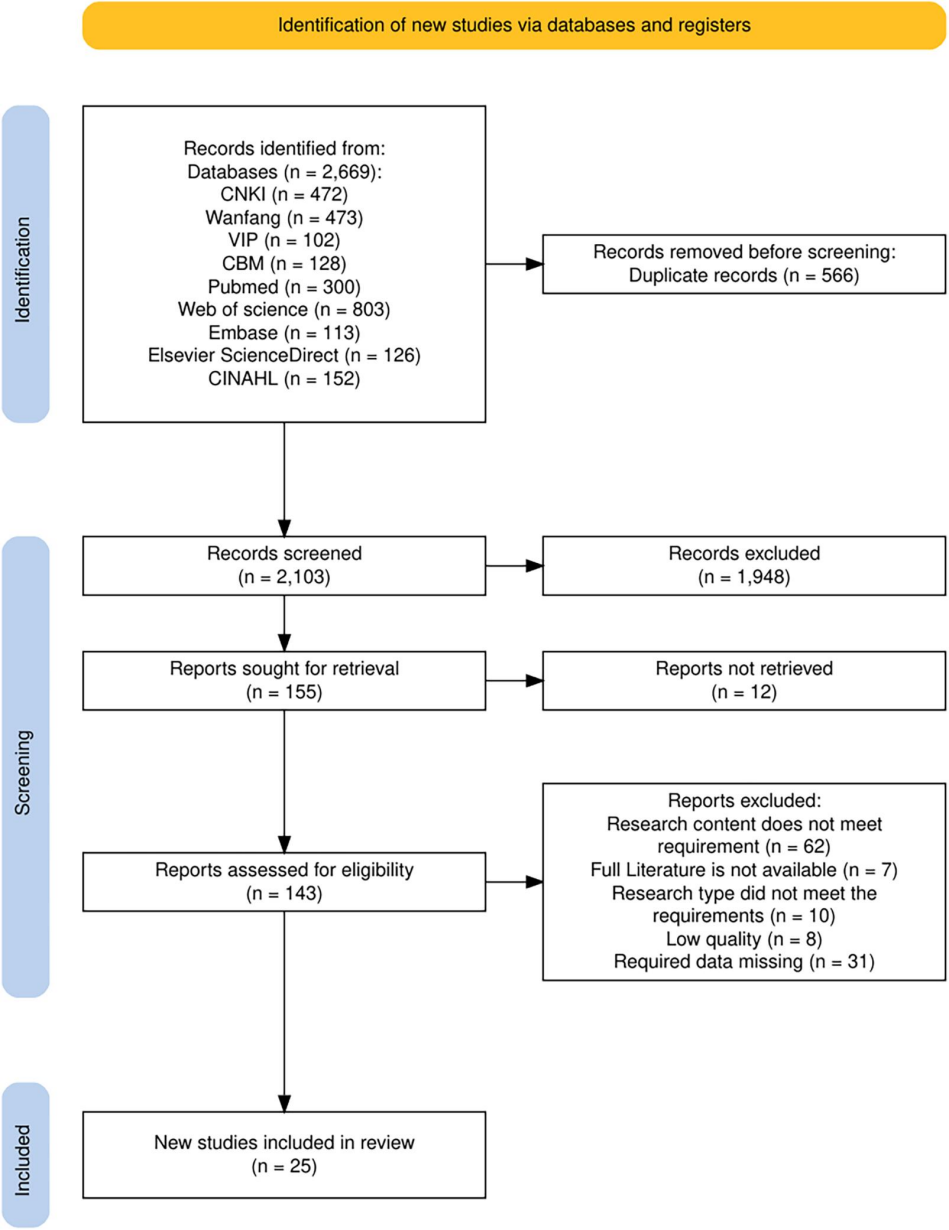
PRISMA flowchart.

**Figure 2:**
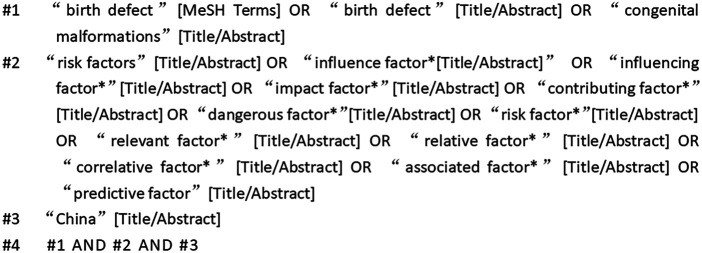
Pubmed search strategy.

Following an initial search, the titles, abstracts, and full texts of the literature were meticulously reviewed and screened according to the established inclusion and exclusion criteria. The quality of the selected literature was subsequently assessed. Ultimately, 25 articles were included in the analysis, encompassing a total of 26,019 cases and 1,097,528 controls. For further details, refer to Figure 1 and Table 1. The PRISMA flowchart is presented in Figure 1, while the PubMed search strategy is illustrated in Figure 2. The search strategy was appropriately adjusted to meet the requirements of each database.

**Table 1:** General characteristics and quality assessment of included studies.

Author	Year	Case	Control	Language	Region	NOS score
Wang et al. (7)	2025	463	7,151	Chinese	Beijing	7
Huang and Li (8)	2025	12	618	Chinese	Shandong	8
Zhou et al. (9)	2024	2,984	1,40,134	English	Hunan	8
Liu et al. (10)	2024	110	490	Chinese	Zhoushan island area	7
Hu et al. (11)	2024	1,951	63,094	Chinese	Qinhuai	8
Zhang et al. (12)	2023	485	23,164	English	Shenyang	7
Zhou (13)	2023	150	150	Chinese	Fujian	7
Zhang et al. (14)	2023	2,060	2,060	Chinese	Chengdu	7
Tian et al. (15)	2023	193	15,493	Chinese	Shandong	8
Li et al. (16)	2023	683	30,851	Chinese	Huangshan	8
Lin (17)	2023	62	432	Chinese	Guangdong	7
Xu et al. (18)	2022	387	17,935	Chinese	Zhejiang	8
Wang et al. (19)	2022	413	413	Chinese	Zhongshan	7
Yang et al. (20)	2021	2,093	66,846	Chinese	Xi An	7
Wang et al. (21)	2021	176	352	Chinese	Zhejiang	7
Li et al. (22)	2021	81	3,502	Chinese	Wenchang	7
Li (23)	2021	29	86	Chinese	NA	7
Zhu et al. (24)	2020	500	500	Chinese	Nanning	8
Zhao (25)	2020	54	681	Chinese	Xinjiang	7
Li et al. (26)	2020	219	219	Chinese	NA	8
Zhu and Ke (27)	2019	387	12,431	Chinese	Quanzhou	7
Wu and Wang (28)	2019	6,080	4,60,803	Chinese	Inner Mongolia	7
Liu et al. (29)	2019	5,386	2,00,817	Chinese	Nanning	8
Liu et al. (30)	2019	941	48,946	Chinese	Guizhou	8
Gao and Li (31)	2019	120	360	Chinese	Shanxi Baoji	8

In the PRISMA flowchart, the search and screening process yielded 25 studies that were included in the analysis. These studies encompassed various types of birth defects, such as congenital heart disease, neural tube defects, cleft lip and palate, among others. Subgroup analyses were conducted based on defect type for risk factors that had sufficient data available.

### 3.2 Heterogeneity test, subgroup analysis, and pooled effect size estimation

Following the heterogeneity testing of the multivariate analysis results for each factor, either random-effects or fixed-effects models were employed for pooling. The findings indicated that premarital examinations, pregnancy examinations, and birth examinations serve as protective factors against birth defects, whereas all other factors were identified as risk factors. Please refer to Table 2, which presents the pooled effect sizes for each factor, along with their corresponding 95% confidence intervals (CIs) and heterogeneity statistics.

**Table 2:** Pooled effect size estimation and heterogeneity test for multivariate analysis of factors influencing birth defects.

Factor	Heterogeneity test		Effect model	Combined OR value	OR 95%CI	*z*	*P*	Document source
	*I*^2^%	*P*						
Maternal age ≥35 years old	0.0%	0.651	Fixed	1.26	1.05–1.47	11.942	<0.001	7 literatures (7, 16, 21, 24–26, 30)
Income < 5,000RMB	60.3%	0.112	Random	1.41	0.90–1.93	5.360	<0.001	2 literatures (18, 30)
Preterm birth	98.9%	<0.001	Random	1.07	0.04–2.10	2.038	0.042	6 (11, 12, 15, 20, 28, 30)
Baby small for gestational age	74.4%	0.020	Random	1.38	0.74–2.01	4.264	<0.001	2 literatures (11, 30)
Adverse pregnancy history	81.3%	<0.001	Random	1.03	0.58–1.48	4.486	<0.001	9 (12–15, 18–22)
Premarital check-up	0.0%	0.726	Fixed	0.36	0.30–0.43	11.243	<0.001	3 literatures (30–32)
Pregnancy test	79.0%	<0.001	Random	0.41	0.21–0.62	3.951	<0.001	6 literatures (15, 24, 25, 30–32)
Antenatal check-up	0.0%	0.939	Fixed	0.45	0.28–0.62	5.109	<0.001	2 literatures (24, 30)
Medication history in early pregnancy	73.8%	0.004	Random	1.17	0.65–1.69	4.386	<0.001	5 literatures (21, 23, 27, 30, 31)
History of fever in early pregnancy	82.0%	<0.001	Random	1.19	0.86–1.52	7.040	<0.001	5 literatures (7, 11, 19, 27, 28)
Adverse mood during pregnancy	89.1%	<0.001	Random	1.09	0.17–2.01	2.315	0.021	4 (8, 23, 30, 31)
Exposure to harmful substances during pregnancy	0.0%	0.442	Fixed	1.45	1.04–1.87	6.859	<0.001	4 literatures (8, 21, 23, 30)
Irregular folic acid supplementation during pregnancy	96.4%	<0.001	Random	1.32	0.09–2.55	2.098	0.036	4 (7, 10, 11, 13)
Take contraceptives 6 months before pregnancy	0.0%	0.401	Fixed	1.36	1.29–1.44	36.006	<0.001	2 literatures (19, 28)
Pregnant women with BMI > 30	0.0%	0.492	Fixed	1.42	1.25–1.58	16.843	<0.001	2 literatures (19, 28)
Mother takes drugs	0.0%	0.809	Fixed	1.06	0.58–1.54	4.328	<0.001	2 literatures (23, 30)
Family history of genetic disease.	0.0%	0.379	Fixed	1.10	0.81–1.39	7.493	<0.001	2 literatures (26, 30)

For factors exhibiting high heterogeneity (*I*^2^ ≥ 50%) and involving at least four included studies—such as adverse pregnancy history, pregnancy examination, preterm birth, early pregnancy medication history, early pregnancy fever history, adverse mood during pregnancy, and irregular folic acid supplementation—subgroup analyses were performed. Among the 25 studies included, the birth defects addressed in the original articles comprised congenital heart disease (10 studies), neural tube defects (6 studies), cleft lip and palate (5 studies), limb defects (3 studies), and other or unspecified defects (8 studies). For factors with adequate data, we performed subgroup analyses based on these types of defects. Detailed subgroup results are presented in Supplementary Table S1. The key findings are summarized below.

Subgroup analysis indicated that when categorized by the type of birth defect, the heterogeneity for most factors was significantly reduced. For instance:

(1) Adverse pregnancy history: The heterogeneity decreased to *I*^2^ = 36.8% in the congenital heart disease subgroup (pooled OR = 1.14, 95% CI: 1.06–1.23).
(2) Preterm birth: The heterogeneity decreased to *I*^2^ = 45.7% in the congenital heart disease subgroup (OR = 1.82, 95% CI: 1.64–2.02) and to *I*^2^ = 0.0% in the neural tube defect subgroup (OR = 1.16, 95% CI: 1.01–1.33).
(3) History of fever in early pregnancy: The heterogeneity decreased to *I*^2^ = 32.5% in the congenital heart disease subgroup (OR = 1.54, 95% CI: 1.31–1.81) and to *I*^2^ = 0.0% in the cleft lip/palate subgroup (OR = 1.38, 95% CI: 1.12–1.70).
(4) Irregular folic acid supplementation: The heterogeneity was completely eliminated in the neural tube defect subgroup (*I*^2^ = 0.0%, OR = 2.34, 95% CI: 2.01–2.72).

In the context of pregnancy examinations, the subgroup analysis for southern China revealed a decrease in heterogeneity to 34.2% (pooled OR = 0.38, 95% CI: 0.30–0.47). Conversely, the heterogeneity for adverse mood during pregnancy and the history of medication in early pregnancy remained high, which may be attributed to differences in exposure definitions and measurement tools utilized across the studies.

### 3.3 Sensitivity analysis

The method of altering the statistical model was employed to evaluate the stability of the meta-analysis results. The findings indicated that, for most factors, the pooled odds ratios (ORs) derived from both the fixed-effects and random-effects models were comparable, with the random-effects model yielding slightly broader confidence intervals. In instances of extreme heterogeneity, such as preterm birth, the two models produced markedly different outcomes, implying that the pooled effect size was significantly influenced by heterogeneity, and that the results of subgroup analyses should be regarded as the primary reference. Overall, the comprehensive analysis of influencing factors in this study is deemed to be generally reliable. Please refer to Table 3, which compares the pooled ORs from both fixed-effects and random-effects models.

**Table 3:** Comparison of pooled OR values from fixed-effects and random-effects models for risk factors of birth defects.

Factor	Fixed effect models incorporate OR values	95%CI	Random effects models incorporate OR values	95%CI
Maternal age ≥35 years old	1.26	1.05–1.47	1.26	1.05–1.47
Income < 5,000RMB	1.51	1.24–1.78	1.41	0.90–1.93
Premature delivery	1.88	1.80–1.96	1.07	0.04–2.10
Baby small for gestational age	1.28	0.97–1.59	1.38	0.74–2.01
Adverse pregnancy history	0.88	0.70–1.06	1.03	0.58–1.48
Premarital check-up	0.36	0.30–0.43	0.36	0.30–0.43
Pregnancy test	0.37	0.28–0.45	0.41	0.21–0.62
Antenatal check-up	0.45	0.28–0.62	0.45	0.28–0.62
Medication history in early pregnancy	1.08	0.82–1.33	1.17	0.65–1.69
History of fever in early pregnancy	1.04	0.96–1.12	1.19	0.86–1.52
Bad mood during pregnancy	0.63	0.37–0.89	1.09	0.17–2.01
Exposure to harmful substances during pregnancy	1.45	1.04–1.87	1.45	1.04–1.87
Not taking folic acid regularly during pregnancy	2.08	1.93–2.23	1.32	0.09–2.55
Take contraceptives 6 months before pregnancy	1.36	1.29–1.44	1.36	1.29–1.44
Pregnant women with BMI > 30	1.42	1.25–1.58	1.42	1.25–1.58
Mother takes drugs	1.06	0.58–1.54	1.06	0.58–1.54
Family history of genetic disease.	1.10	0.81–1.39	1.10	0.81–1.39

### 3.4 Publication bias

Funnel plot methods for testing publication bias typically require a minimum of 10 studies to ensure adequate statistical power. In this study, no factor included more than 10 studies, which precluded the performance of funnel plots and Egger's tests. This limitation highlights a significant constraint in our analysis.

## 4 Discussion

### 4.1 Main findings

This meta-analysis of 25 case-control studies, encompassing 26,019 cases, identified several factors associated with birth defects in China from 2019 to 2025. Protective factors included premarital examinations, pregnancy examinations, and birth examinations. Conversely, risk factors encompassed advanced maternal age, low income, preterm birth, small for gestational age, adverse pregnancy history, medication or fever in early pregnancy, adverse mood, exposure to harmful substances, irregular folic acid supplementation, contraceptive use prior to pregnancy, high maternal body mass index (BMI), maternal drug use, and a family history of genetic disorders. Subgroup analyses indicated that the observed heterogeneity largely arose from differences in defect types, with several factors specific to the Chinese context emerging.

### 4.2 Comparison with previous studies

In comparison to meta-analyses published prior to 2019, our pooled odds ratio (OR) for advanced maternal age (1.26) is slightly elevated compared to earlier estimates (approximately 1.15–1.20), potentially reflecting an increased proportion of women aged 40 years or older in the post-three-child policy era (2, 33). The protective effect of premarital examinations (OR = 0.36) aligns with findings from a 2017 meta-analysis (OR = 0.34), suggesting that this factor has remained consistent over time despite the voluntary policy change in 2003. However, the pooled OR for low income (less than 5,000 RMB/month) in our study (1.41) exceeds that reported in previous Chinese studies (1.20–1.30), which may indicate a worsening of economic disparities in healthcare access in recent years. Additionally, our observation that irregular folic acid supplementation is most strongly associated with neural tube defects (OR = 2.34, *I*^2^ = 0%) corroborates and reinforces the well-established causal relationship (42), while the absence of heterogeneity in this subgroup suggests a robust association across various populations.

### 4.3 China-specific factors and novel findings from subgroup analyses

Several findings are particularly pertinent to China's current context and represent novel contributions of this study.

First, premarital examination (OR = 0.36) remains a significant protective factor, even after its transition to a voluntary status in 2003. The low uptake of premarital exams in certain regions, with reported rates as low as 30%–40%, indicates a pressing need for targeted public health campaigns aimed at increasing voluntary participation, particularly in rural areas.

Second, the regional heterogeneity observed in the protective effect of pregnancy examinations (southern China: *I*^2^ = 34.2%, pooled OR = 0.38; northern China: *I*^2^ = 56.3%, OR = 0.45) highlights the disparities in healthcare quality and access across China. Southern provinces typically exhibit higher GDP per capita and more comprehensive maternal health services, which may account for the more consistent and slightly stronger protective effect observed in these regions.

Third, defect-type specific associations that have rarely been reported in previous Chinese meta-analyses were identified:

(1) Preterm birth demonstrated the strongest association with congenital heart disease (OR = 1.82) and cleft lip/palate (OR = 1.41), but not with neural tube defects (OR = 1.16, with the 95% CI including 1.0 in some analyses). This suggests that the pathological mechanisms linking preterm birth to different defect types may vary, potentially involving hemodynamic changes during cardiac development as opposed to independent genetic pathways.
(2) Irregular folic acid supplementation was exclusively and strongly associated with neural tube defects (OR = 2.34, *I*^2^ = 0%), while its association with other defects (e.g., congenital heart disease, cleft lip/palate) was weaker and exhibited high heterogeneity. This finding reinforces the specificity of folic acid in preventing neural tube defects, which is a crucial message for China's national folic acid supplementation program.

Fourth, an adverse pregnancy history, including previous stillbirth, miscarriage, or neonatal death, was significantly associated with congenital heart disease (OR = 1.14) and cleft lip/palate (OR = 1.21) following subgroup analysis. This indicates that the recurrence risk is specific to the type of defect. These findings support the need for targeted genetic counseling for couples with a history of specific defects. Furthermore, the defect-type specific results highlight the necessity of moving beyond a one-size-fits-all approach in birth defect research. Future studies should report risk factors separately for each major defect category.

### 4.4 Interpretation of key risk factors

Maternal physiological factors: Mothers aged 35 years and older are at a higher risk of having offspring with birth defects. Compared to women of non-advanced maternal age, those of advanced maternal age exhibit a greater incidence of pregnancy complications, fetal chromosomal abnormalities, and birth defects. Research has indicated that increased rates of oocyte aneuploidy contribute to elevated risks of infertility, miscarriage, and adverse pregnancy outcomes, including birth defects, in women of advanced maternal age (33). A maternal body mass index (BMI) exceeding 30 kg/m^2^ during pregnancy signifies inadequate weight management and is correlated with an increased risk of pregnancy complications. Consequently, in light of the prevailing trend of advancing maternal age, it is imperative to enhance prenatal screening and diagnostic services for pregnant women of advanced maternal age. Additionally, maternal drug use significantly heightens the likelihood of birth defects in offspring. Studies have demonstrated (34, 35) that drugs can traverse the placenta from maternal circulation to the fetus, resulting in toxic effects.

Physiological and Health Status During Pregnancy: The first 3–8 weeks after conception, known as the first trimester, is a critical period for embryonic development, during which fetal organ formation and development are particularly sensitive. Fever and medication use may disrupt normal physiological processes in the fetus, leading to abnormalities in cell division and differentiation, thus increasing the risk of fetal malformations. Studies have indicated that fever during early pregnancy may be associated with congenital heart disease (36), cleft lip and palate (37), cryptorchidism (38), and other birth defects. Furthermore, medication use during this period has been linked to cleft lip and palate (39), congenital pulmonary airway malformation (40), and external auditory canal malformations (41). During pregnancy, it is also crucial to monitor the mother's psychological status, as adverse moods may elevate the risk of birth defects, potentially due to hormonal changes associated with emotional fluctuations. Additionally, exposure to harmful substances during pregnancy can negatively impact fetal health.

Use of Nutritional Supplements: Irregular folic acid supplementation during pregnancy may lead to birth defects. As a water-soluble B vitamin, folic acid is crucial for fetal growth and development, as well as for maternal health during pregnancy. It effectively prevents various birth defects, including neural tube defects, congenital heart disease, and cleft lip and palate, while promoting fetal cell division and growth, thereby ensuring normal organ formation (42). Irregular folic acid supplementation during pregnancy may result in insufficient folic acid intake, consequently increasing the risk of birth defects. Furthermore, folic acid deficiency may adversely affect the uterine environment, heightening the likelihood of preterm birth and negatively impacting fetal intellectual development. Therefore, pregnant women should supplement folic acid in a reasonable and regular manner under medical guidance. Additionally, they should undergo regular prenatal check-ups to monitor folic acid levels and ensure optimal supplementation.

Healthcare Service Factors: Premarital examinations, pregnancy check-ups, and birth examinations can timely identify risk factors and detect birth defects early, thereby gaining valuable time for implementing corresponding intervention measures. The regional heterogeneity observed suggests that improving access to and the quality of prenatal care in northern and western China should be a policy priority.

### 4.5 Clinical and policy implications

Based on our findings, we recommend the following:

1. Strengthen folic acid supplementation programs with a focus on the periconceptional period, especially for women planning pregnancy in rural areas where irregular use is common.
2. Revitalize premarital examination campaigns using voluntary but incentivized models (e.g., free or low-cost screening packages).
3. Target advanced maternal age women (≥35 years) with enhanced prenatal screening and genetic counseling.
4. Address regional disparities in prenatal care quality, particularly in northern and western provinces, by standardizing pregnancy examination protocols.
5. For women with adverse pregnancy history or family history of genetic disorders, provide defect-type specific counseling and monitoring.

## 5 Limitations

This study presents several limitations. First, the majority of the included studies were conducted in eastern or southern China, which restricts the generalizability of the findings to western and northern regions. Second, the case-control designs employed are susceptible to recall bias, particularly concerning maternal behaviors during pregnancy (e.g., folic acid supplementation and medication use). Third, while all included Chinese-language studies met our Newcastle-Ottawa Scale (NOS) quality threshold (≥4), they may possess inherent limitations, including incomplete reporting of exposure definitions, a lack of standardized outcome assessments, and potential publication bias favoring positive results. Fourth, some subgroups (e.g., cleft lip and palate, neural tube defects) included only 2–3 studies, making the pooled results exploratory in nature. Fifth, no factor had 10 or more studies, which precluded formal tests for publication bias. Lastly, the definitions of “adverse mood during pregnancy” varied significantly across studies, with differences in scales, cutoff values, and timing. Moreover, none of the original studies specified the measurement tools utilized, which may have contributed to persistent heterogeneity and highlights a methodological gap in this field. Future research should employ validated scales (e.g., SAS, SDS, HADS) with clearly defined timing and cutoff values.

## 6 Conclusion

Numerous factors influence the prevalence of birth defects in China. It is imperative for medical professionals to prioritize irregular folic acid supplementation, low premarital examination rates, and regional disparities in prenatal care as primary targets for intervention. Implementing targeted strategies to address controllable factors is essential for the prevention and reduction of birth defects. Future research should concentrate on risk factors specific to different types of defects, utilize standardized exposure assessment tools, and incorporate more representative samples from northern and western regions of China.
