# Supplementary material for: Maternal, healthcare, and nutritional factors influencing birth defects in China from 2019 to 2025: a systematic review and meta-analysis
Source: Front Pediatr. 2026 Jul 3;14:1842814. doi: 10.3389/fped.2026.1842814 (PMC13377976; doi:10.3389/fped.2026.1842814)
Supplement: Supplementary file 1 [file Table1.doc]

Supplementary table 1 Subgroup analysis results for factors with high heterogeneity

| **Factor** | **Subgroup** | **Number of Studies** | **I² (%)** | **Effect Model** | **Pooled OR** | **95% CI** | **P (pooled)** |
| --- | --- | --- | --- | --- | --- | --- | --- |
| ****Adverse pregnancy history**** | Congenital heart disease | 4 | 36.8 | Fixed | 1.14 | 1.06–1.23 | <0.001 |
|  | Neural tube defects | 3 | 41.2 | Fixed | 1.09 | 0.98–1.21 | 0.112 |
|  | Cleft lip and palate | 2 | 0.0 | Fixed | 1.21 | 1.03–1.42 | 0.019 |
|  | Other/unclassified | 4 | 68.5 | Random | 0.96 | 0.72–1.28 | 0.785 |
| ****Pregnancy examination**** | Southern China | 4 | 34.2 | Fixed | 0.38 | 0.30–0.47 | <0.001 |
|  | Northern China | 2 | 56.3 | Random | 0.45 | 0.32–0.59 | <0.001 |
| ****Preterm birth**** | Congenital heart disease | 3 | 45.7 | Fixed | 1.82 | 1.64–2.02 | <0.001 |
|  | Neural tube defects | 2 | 0.0 | Fixed | 1.16 | 1.01–1.33 | 0.035 |
|  | Cleft lip and palate | 2 | 38.9 | Fixed | 1.41 | 1.20–1.66 | <0.001 |
|  | Other/unclassified | 3 | 92.3 | Random | 0.89 | 0.51–1.55 | 0.682 |
| ****History of medication in early pregnancy**** | Southern China | 3 | 58.2 | Random | 1.21 | 0.94–1.56 | 0.140 |
|  | Northern China | 2 | 0.0 | Fixed | 1.09 | 0.82–1.45 | 0.545 |
| ****History of fever in early pregnancy**** | Congenital heart disease | 3 | 32.5 | Fixed | 1.54 | 1.31–1.81 | <0.001 |
|  | Cleft lip and palate | 2 | 0.0 | Fixed | 1.38 | 1.12–1.70 | 0.002 |
|  | Neural tube defects | 2 | 0.0 | Fixed | 1.12 | 0.89–1.41 | 0.331 |
|  | Other/unclassified | 3 | 78.6 | Random | 1.03 | 0.76–1.39 | 0.842 |
| ****Adverse mood during pregnancy**** | NOS score 7 | 3 | 82.4 | Random | 1.21 | 0.87–1.68 | 0.259 |
|  | NOS score 8 | 1 | / | / | 1.04 | 0.65–1.67 | 0.871 |
| ****Irregular folic acid supplementation**** | Neural tube defects | 2 | 0.0 | Fixed | 2.34 | 2.01–2.72 | <0.001 |
|  | Other defects | 2 | 87.5 | Random | 1.12 | 0.69–1.82 | 0.648 |
